# A Bioinformatics Perspective on the Links Between Tetraspanin-Enriched Microdomains and Cardiovascular Pathophysiology

**DOI:** 10.3389/fcvm.2021.630471

**Published:** 2021-03-30

**Authors:** Ge Sun, Junxiong Chen, Yingjun Ding, Jonathan D. Wren, Fuyi Xu, Lu Lu, Yan Wang, Dao-wen Wang, Xin A. Zhang

**Affiliations:** ^1^University of Oklahoma Health Sciences Center, Oklahoma City, OK, United States; ^2^Oklahoma Medical Research Foundation, Oklahoma City, OK, United States; ^3^University of Tennessee Health Science Center, Memphis, TN, United States; ^4^Tongji Medical College of Huazhong University of Science and Technology, Wuhan, China

**Keywords:** angiogenesis, atherosclerosis, blood pressure, integrin, platelet

## Abstract

**Background:** Tetraspanins and integrins are integral membrane proteins. Tetraspanins interact with integrins to modulate the dynamics of adhesion, migration, proliferation, and signaling in the form of membrane domains called tetraspanin-enriched microdomains (TEMs). TEMs also contain other cell adhesion proteins like immunoglobulin superfamily (IgSF) proteins and claudins. Cardiovascular functions of these TEM proteins have emerged and remain to be further revealed.

**Objectives:** The aims of this study are to explore the roles of these TEM proteins in the cardiovascular system using bioinformatics tools and databases and to highlight the TEM proteins that may functionally associate with cardiovascular physiology and pathology.

**Methods:** For human samples, three databases—GTEx, NCBI-dbGaP, and NCBI-GEO—were used for the analyses. The dbGaP database was used for GWAS analysis to determine the association between target genes and human phenotypes. GEO is an NCBI public repository that archives genomics data. GTEx was used for the analyses of tissue-specific mRNA expression levels and eQTL. For murine samples, GeneNetwork was used to find gene–phenotype correlations and gene–gene correlations of expression levels in mice. The analysis of cardiovascular data was the focus of this study.

**Results:** Some integrins and tetraspanins, such as *ITGA8* and *Cd151*, are highly expressed in the human cardiovascular system. TEM components are associated with multiple cardiovascular pathophysiological events in humans. GWAS and GEO analyses showed that human *Cd82* and *ITGA9* are associated with blood pressure. Data from mice also suggest that various cardiovascular phenotypes are correlated with integrins and tetraspanins. For instance, *Cd82* and *ITGA9*, again, have correlations with blood pressure in mice.

**Conclusion:** *ITGA9* is related to blood pressure in both species. KEGG analysis also linked *ITGA9* to metabolism and MAPK signaling pathway. This work provides an example of using integrated bioinformatics approaches across different species to identify the connections of structurally and/or functionally related molecules to certain categories of diseases.

## Introduction

### Tetraspanin-Enriched Microdomains

**Figure 1:**
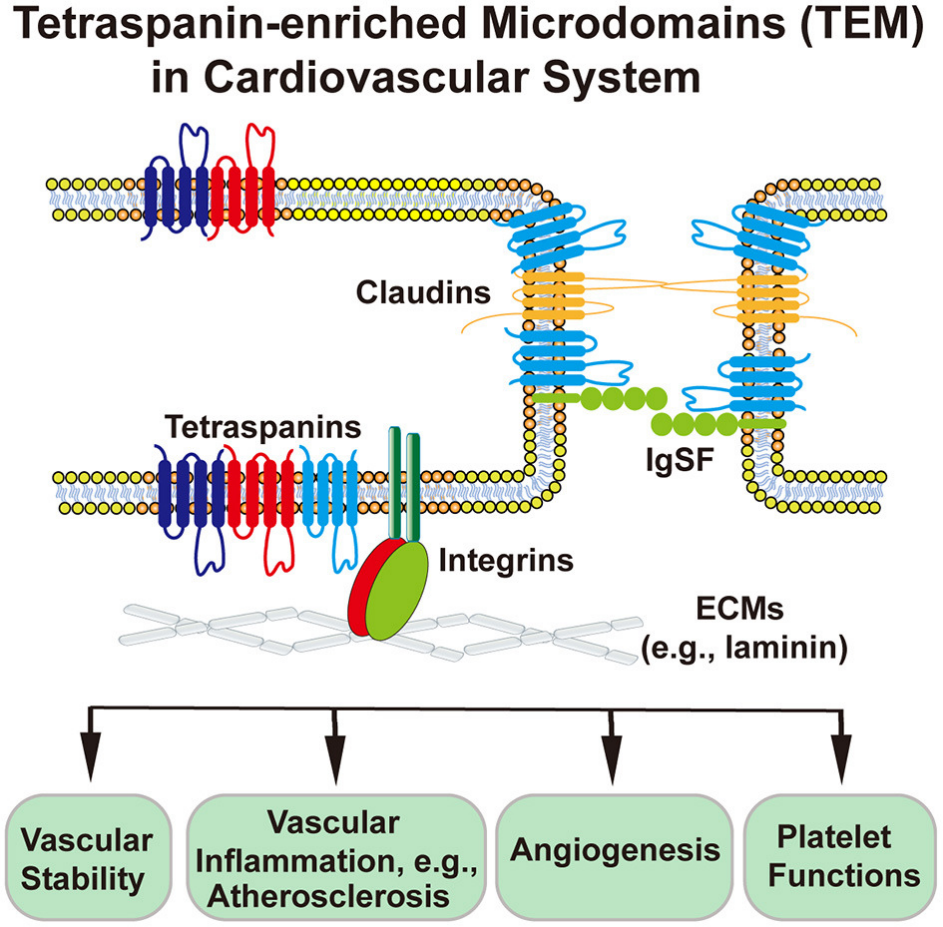
Schematic presentation of tetraspanin-enriched microdomains (TEMs) at the cell–ECM and cell–cell interfaces and TEM-involved or TEM-regulated cardiovascular functions. Endothelial cell is used as an example of cardiovascular cells. In endothelial cells, tetraspanins associate with each other and with integrins, claudins, and IgSF proteins, to regulate physiological and pathological activities of heat, vessel, and blood.

Tetraspanin-enriched microdomains (TEMs) are ubiquitously present in a variety of cells such as endothelial cells (ECs), vascular smooth muscle cells (VSMCs), leukocytes, and platelets. Mainly formed by tetraspanins, TEMs also contain other membrane proteins such as integrins, immunoglobulin superfamily (IgSF) proteins, and claudins (1) (Figure 1).

Besides four membrane-spanning segments, each tetraspanin has a highly conserved CCG motif in large extracellular loop and a few polar residues in transmembrane domains (2). The functions of the 33 human tetraspanins are quite diverse, with unknown underlying general mechanism. Integrins are heterodimeric receptors mainly for extracellular matrices (ECMs), conducting both inside-out and outside-in signaling for various cellular functions (3) such as cell adhesion, migration, proliferation, and survival (4, 5).

### Recent Progress in Vascular Pathophysiology of TEMs

#### Tetraspanins

Studies on tetraspanins in blood vessels were largely limited to CD9, CD63, CD151, and Tspan12 (6, 7). A recent study revealed that CD82 restrains pathological angiogenesis by facilitating cell adhesion protein endocytosis and then confining EC movement (8). The miR-K6-5p of Kaposi's sarcoma-associated herpesvirus represses CD82 expression by targeting its coding sequence and promotes EC invasion and angiogenesis by activating c-Met signaling (9). Postnatal angiogenesis becomes severely impaired in tetraspanin *Tspan8-null* and *Cd151-null; Tspan8-null* mice, together with reduced EC–ECM adhesion and EC migration upon *Cd151* and *Tspan8* ablations (10), resembling the vascular impacts of *Cd151-null* mice reported earlier. Tspan8 appears to support the lymphangiogenesis induced by pancreatic cancer. CD151-integrin α6β1 complexes drive embryonic stem cells on Laminin 111 to differentiate to ECs, but CD151–integrin α3β1 complexes do not (11). Tetraspanin Tspan18 is expressed in developing vasculature and primary ECs, and its silencing via morpholino in zebrafish leads to vascular defects in angiogenesis, vessel stability, and arterial-venous specification through vascular endothelial growth factor (VEGF)/VEGFR and Notch signaling (12). VEGF-induced expression of tm4sf18, a tetraspanin-related four-transmembrane protein, in ECs serves as a positive feedback mechanism to amplify VEGF signaling and therefore enhance angiogenesis, in contrast to the well-established negative feedback mechanism of Notch in VEGF-dependent angiogenesis (13).

A recent study showed that CD9 antibody or *Cd9* ablation attenuates the atherosclerotic plaque progression in mouse as CD9 promotes senescence of ECs (14). Consistently, CD9 expression becomes increased in human arteries upon aging and in atherosclerotic arteries in human (15). CD151 sustains EC–EC and EC–ECM adhesions and confines cytoskeletal tension within ECs, to support vascular stability (16). Differential expressions of Tspan7 in ischemic and non-ischemic muscles between C57BL/6 and BALB/c mouse strains, which display differential susceptibility to hindlimb ischemia and were used to identify the genes associated with vascular inflammatory responses to hindlimb ischemia, suggest a regulatory role of Tspan7 in peripheral arterial disease (17). Given that tetraspanins serve not only as markers but also as regulators of exosomes, direct participation of the exosomes from VSMCs in artery calcification (13) suggests tetraspanin involvement in this vascular event. Tspan2 may be associated with VSMC differentiation as its selective expression in VSMCs is dramatically upregulated by TGFβ or myocardin, two activators of VSMC differentiation, but becomes downregulated in mouse carotid arteries after ligation injury and in human arteriovenous fistula samples after occlusion by dedifferentiated neointimal VSMCs (18). Human-induced pluripotent stem cell (hiPSC)-derived cardiomyocyte progenitors that express CD82 almost exclusively differentiate into cardiomyocytes both *in vitro* and *in vivo*, and CD82 is involved in fate commitment to cardiomyocytes through Wnt signaling inhibition (19). Hence, CD82 may serve as a marker for prospectively isolating cardiomyocyte progenitors.

Mutations in *TSPAN12* cause familial exudative vitreoretinopathy (FEVR) (20, 21). As a co-receptor of Norrin, Tspan12 interacts with Norrin and its receptor Frizzled-4 via its large extracellular loop and enhances the selectivity of Norrin binding to Frizzled-4, while FEVR-linked mutations in *TSPAN12* disrupt the co-receptor function of Tspan12 (22). Besides its importance in retinal vasculature development, Tspan12 in endothelia is also needed for the maintenance of the blood–retina barrier (23). As an exciting advancement, Tspan12 antibody displayed therapeutic benefits for vasoproliferative retinopathies in mouse models by limiting β-catenin signaling, and a combination of the antibody and VEGF antagonist markedly improved the efficacy of VEGF antagonist (24). Similarly, norrin restores diabetes-caused or VEGF-induced disruption of the blood–retina barrier by enhancing tight junction and through β-catenin (25). Intriguingly, VEGF facilitates norrin signaling by elevating the presence of Tspan12 at the EC surface in a MAPK signaling-dependent manner. Tspan12 also supports norrin signaling for the maintenance of the blood–brain barrier (26).

In contrast to increased bleeding upon murine *Cd151* ablation, *Cd82* ablation reduces bleed time in mouse by enhancing integrin αIIbβ3 expression on platelets and clot retraction (27). Both CD151 and tetraspanin TSSC6 are physically associated with ADP purinergic receptor P2Y12 and functionally complement P2Y12 roles in platelet aggregation and thrombus stabilization (28, 29). Moreover, Tspan18 is required for the proper formation of thrombi in response to inflammatory stimuli and deposition of platelets in response to vascular injury by promoting Orai1/Ca^2+^ signaling in and von Willebrand factor release from ECs (30). CD63 is required for stabilizing P-selectin at the EC surface to recruit leukocytes (31), and annexin A8 facilitates trafficking of CD63 from late endosome to Weibel–Palade body and EC surface and stabilizes the presence of CD63 and P-selectin on the EC surface for leukocyte recruitment (32).

#### IgSF Proteins and Claudins

IgSF proteins EWI-2 and EWI-F associate physically with tetraspanins CD9, CD81, and CD82 (1, 33) (Figure 1). EWI-2 connects tetraspanins to actin cytoskeleton, interacts with phospholipids with its cytoplasmic domain (34, 35), and associates indirectly with some integrins via tetraspanins (36, 37). Claudins 1, 7, and 11 are associated with tetraspanins CD9, CD81, Tspan8, and Tspan3 (Figure 1). As claudins are essential constituents of tight junction, TEMs to which claudins partition in ECs are predicted to regulate the function of endothelial barriers such as the blood–brain barrier and the blood–retina barrier (38).

#### Integrins

Besides being successful therapeutic targets of thrombosis, integrins are emerging targets for other cardiovascular diseases, especially atherosclerosis (39), due to their crucial functions in platelets and ECs (40, 41). Integrin α*νβ*3 is used as an angiogenesis marker for early-stage atherosclerosis imaging (42), in addition to (i) its supporting role in tumor and other pathological angiogenesis such as Matrigel plug and aortic ring angiogenesis and (ii) its potential as a therapeutic target against tumor angiogenesis (43–45). Interestingly, exosomal integrin αvβ6 released by prostate cancer cells promotes vascular morphogenesis, probably by confining STAT1 phosphorylation (46). Integrin β3 was also recently shown to regulate VSMCs during atherogenesis (47). Other integrins were found to be important in blood vessel maturation (48) and stability (49). For example, heterozygous ablation of endothelial integrin β1 or inhibition of integrin β1 by its function-blocking antibody reduced acute vascular leakage caused by bacterial endotoxin (50). Integrins in VSMCs regulate the contractile function and affect arterial stiffening (51), while blockades of integrin α5β1–fibronectin and α5β1–fibrin interactions enhance the calcification of valvular interstitial cell of cardiac valves (52). In addition to ECs and VSMCs, integrins in leukocytes, mainly β1 and β2 integrins, are well-documented for their roles in vascular inflammatory response (53). Recent advances reveal that sharp upregulation of integrin α3β1 in neutrophils during sepsis is associated with Toll-like receptor-induced vascular inflammatory responses and cytokine productions (54) and that neutrophils aid oxidation-dependent modification of extracellular matrix during vascular inflammation and then the oxidation promotes integrins α_M_β_2_- and α_D_β_2_-mediated vascular infiltration of macrophages (55). Apparently, there is still much to learn about the roles of integrins in the pathogenesis of cardiovascular diseases (56, 57), for the purpose of disease treatment.

To expand our knowledge about TEMs (Figure 1), we combine multiple bioinformatics tools and databases to investigate putative linkages of each member of the tetraspanin and integrin superfamilies to cardiovascular events systematically. Bioinformatics proves beneficial when it comes to predicting the disease risk factors and targets.

## Methods

### Databases With Information From Human Samples

#### The Genotype-Tissue Expression Database

The Genotype-Tissue Expression (GTEx) database provides human gene expression levels and their regulatory relationships to genetic variations, stressing the concept of expression quantitative trait loci (eQTL). This database was used to gather the mRNA levels of all integrins and tetraspanins. Version 8 of GTEx, which includes 17,382 samples from 948 human donors, was downloaded on January 2021 at https://gtexportal.org/.

#### The Database of Genotypes and Phenotypes

The Database of Genotypes and Phenotypes (dbGaP) is a National Center for Biotechnology Information (NCBI)-affiliated database investigating the associations between human genotypes and phenotypes (58). The results from genome-wide association studies (GWAS) housed in National Human Genome Research Institute were merged with the other genetic databases from NCBI, such as Gene and dbSNP, to suggest potential genotype–phenotype correlations that are more general than the typical mutation–disease linkage. We used the public data interface Phenotype–Genotype Integrator (PheGenI) (59) to detect every gene belonging to the two functionally related families for their associations with cardiovascular phenotypes. Phenotypes such as platelet function and cholesterol were also included for their relevance to coronary heart disease and thrombosis. All deposited datasets (1,232 in total) in this database were included in this study. The data was downloaded in November 2018 at https://www.ncbi.nlm.nih.gov/gap/. Linear regression or logistic regression was used for statistical analysis.

#### Gene Expression Omnibus for Literature Mining and Transcriptional Correlation Network Construction

Gene Expression Omnibus (GEO) is an NCBI database that deposits a wide range of high-throughput genomics data across species and under numerous normal or diseased conditions. We utilized all human datasets to predict the correlated genes of our molecules of interest.

The literature-mining approach IRIDESCENT (60–62) was used to map relations between genes, diseases, and phenotypes. Briefly, it uses a thesaurus of terms, including spelling and abbreviation variants, to identify when they co-occur within PubMed abstracts. The network of relationships can then be interrogated for commonalities. The latest version was run on MEDLINE on January 2021, which included 31,907,626 total records.

For GAMMA analysis, data for the microarray platform GPL570 was downloaded in August 2018 from NCBI's GEO repository (https://www.ncbi.nlm.nih.gov/geo/), consisting of 80,600 samples from 2,698 experiments. Sample data (GSM files) was quantile normalized prior to calculating a matrix of gene–gene correlations using Pearson's correlation coefficient (*r*) (ALGLIB, Sergey Bochkanov, 2007, https://www.alglib.net/). Using the *K*-nearest neighbors (KNN) approach (*K* = 40), a set of the most highly correlated genes was obtained for each gene as described previously (63). Using a “guilt by association” approach, literature-based commonalities were identified for the most highly correlated genes with IRIDESCENT (62). The result is a list of literature-based enrichments for the correlated genes (63) that are, in turn, predicted functions for individual genes. The categories of commonalities identified are diseases, phenotypes, genes, chemicals, drugs, and Gene Ontology categories. GAMMA identifies the top 40 most correlated genes for each input gene, and then IRIDESCENT provides an analysis of what those genes have in common in MEDLINE in terms of (1) the name of the connected concept or commonality (e.g., diseases, genes, etc), (2) the number of genes (out of 40) connected to the concept in MEDLINE or shared relationships, and (3) an observed to expected value that reflects how many connections were observed relative to the number expected by chance, given the number of connections of each concept in the analysis. The “Score” reflects a balance between how unusual the observed number of connections to a concept is, given how connected each object is in the subnetwork being analyzed, and how many connections were made. If only the observed to expected (Obs/Exp) value is used to score connections between concepts, the highest values tend to be between relatively rare concepts. These could be potentially interesting, but at the same time, the association is based upon less evidence. Conversely, the most connected concepts are almost always the most abundant concepts in general. Thus, the score provides a way to balance the search for a statistically unusual number of links between concepts with the amount of evidence available.

### Databases With Information From Mouse Samples

#### GeneNetwork

The GeneNetwork website (http://genenetwork.org/) (64) provides genomic data from various mouse strains and different murine tissue samples. The main purpose of this database is to investigate genome-to-phenome relationships in mice (65). We used this database to (i) associate integrins and tetraspanins with mouse cardiovascular phenotypes; (ii) find correlations between expression levels of different genes, specifically in mouse aorta samples; and (iii) predict tissue-specific signaling pathways of a protein of interest by the gene–gene associations. The mouse aorta transcriptome data [UCLA BXD Aorta Affy M430 2.0 (Jan16) RMA] and cardiovascular phenotype data were downloaded on December 2019 from GeneNetwork and used for Pearson correlation coefficient analysis to identify gene–phenotype and gene–gene associations, with the resulted *p* < 0.05 as the significance threshold. The analysis was carried out using R package v4.03 (66), and the aorta transcriptome data were deposited to and can be accessed on GeneNetwork and GEO under the accession numbers of GN819 and GSE66569, respectively. A total of 38 mouse lines, consisting of 36 BXD strains and two C57BL/6J and DBA2/J parental strains, were included in the aorta transcriptome data analysis. Gene set enrichment analysis was performed at the Webgestalt website (http://www.webgestalt.org) (67), to investigate the Kyoto Encyclopedia of Genes and Genomes (KEGG) pathway. The *p* value generated from the test was automatically adjusted to account for multiple comparisons using the Benjamini and Hochberg correction (68). A minimum overlap of five genes and an adjusted *p* < 0.05 were required to determine the genes significantly overrepresented in those categories.

## Results

### Some Integrins and Tetraspanins, Such as ITGA8 and Cd151, Are Highly Distributed in the Human Cardiovascular System

**Figure 2:**
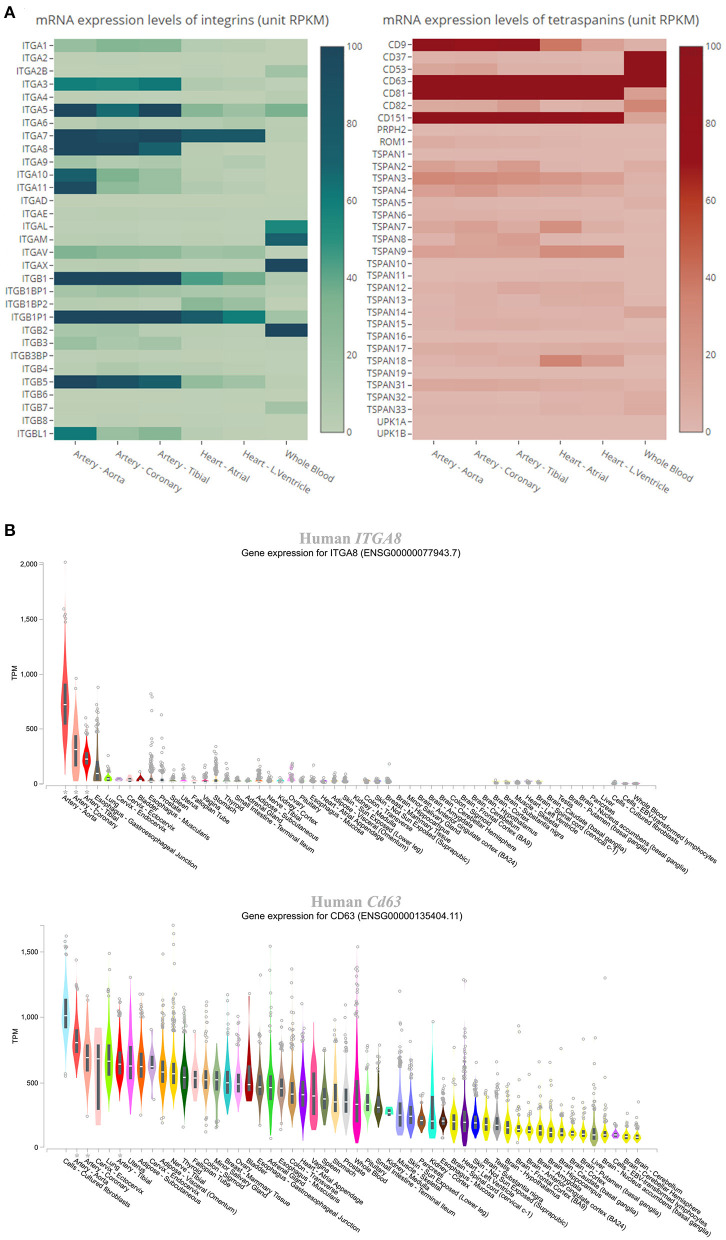
Expression levels of integrins and tetraspanins in human cardiovascular tissues. **(A)** Heatmaps showing the mRNA levels in the units of RPKM (reads per kilobase of transcript, per million mapped reads) of the integrins and tetraspanins in human aorta, coronary artery, tibial artery, heart atrium, left ventricle, and whole blood. **(B)** Box plot showing the expression levels, presented as TPM (transcripts per kilobase million), of *ITGA8* and *Cd63* across various human tissues. All data were extracted from GTEx.

The expression level of each gene of tetraspanins and integrins in the human cardiovascular system was determined by GTEx. According to the data, as shown in the heatmaps (Figure 2A), the integrin and tetraspanin superfamilies have contrast compositions in the cardiovascular system, suggesting a possible discrepancy or divergence in their functions. The mRNA levels of integrins α3, α5, α7, α8, αV, β1, and β5 and tetraspanins TSPAN3, TSPAN4, TSPAN9, CD9, CD63, CD81, and CD151 range from moderate to high. In mouse aortas, tetraspanins CD151, CD81, TSPAN3, and CD9; integrins β5, β1, and αV; and integrin-associated protein Fermt2/Kindlin2 are relatively highly expressed (Table 1), although this study/dataset did not dissect the distributions of these expressions in EC and/or VSMC compartments of the aortas. Given that VSMCs constitute the predominant cell mass in aorta, these expressions more likely represent the expressions from the VSMCs of aorta.

**Table 1:** Mouse integrins, tetraspanins, and integrin-associated proteins with the gene expression levels that are significantly higher than the median expression level of all genes in mouse aortas.

**ProbeSet ID**	**Symbol**	**Gene ID**	**Mean expression level (modified *Z*-score)**
1456085_x_at	*Cd151*	12476	13.71881579
1416330_at	*Cd81*	12520	13.46648421
1434180_at	*Fermt2*	218952	12.60605263
1454966_at	*Itga8*	241226	12.19214211
1416009_at	*Tspan3*	56434	12.10072895
1417533_a_at	*Itgb5*	16419	11.92098684
1452545_a_at	*Itgb1*	16412	11.83133947
1416066_at	*Cd9*	12527	11.78268421
1452784_at	*Itgav*	16410	11.52730263

*Data were obtained from GeneNetwork. The expression levels were standardized using the 2z+8 method, transferred by the log2 scale, and presented as modified Z-scores in which a 2-fold difference in expression corresponds approximately to a 1-unit change, as described in the methods and in Bennett et al. (69) and Chesler et al. (70)*.

The genes that are abundant in both human and mouse cardiovascular systems were chosen for comparisons of their expression levels across various human tissues, using data from GTEx. All of the genes are highly expressed at the mRNA level in arteries, including the aorta, coronary arteries, and tibial arteries. Figure 2B shows genes *ITGA8* and *Cd63*, which are highly expressed in the arteries, as examples for integrin and tetraspanin families, respectively. We must acknowledge that, as human tissue specimen examined in GTEx largely came from healthy subjects, the low expressions for some of these tetraspanin and integrin genes at physiological condition do not dismiss the possibility of upregulation of their expressions under pathological conditions. For other organs such as the liver and lung, their expressions in the vasculature within the organs are difficult to be determined.

### GWAS Shows Associations Between Integrins or Tetraspanins and Cardiovascular Events

**Figure 3:**
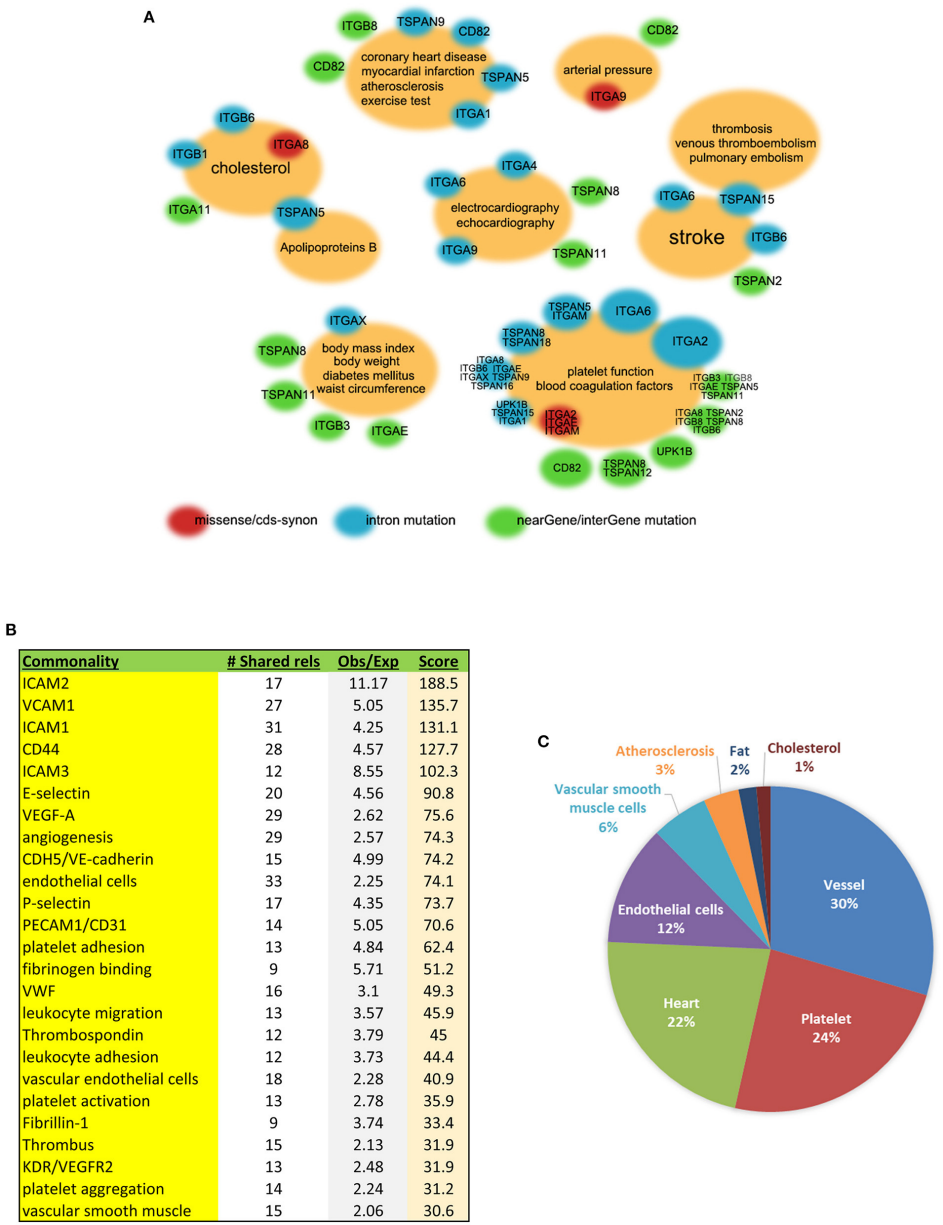
Integrin and/or tetraspanin genes associated with multiple cardiovascular phenotypes in humans. **(A)** Association clouds with GWAS phenotypes at the centers and related genes at the surrounding. Small red clouds with gene names denote an association between a missense and/or cds-syn mutation(s) of the genes and the phenotype(s) in the center; blue denotes intron mutation; green denotes the 3′ or 5′ untranslated region, near-gene, or intergene SNPs. The size of the surrounding clouds represents the number of associations. Data from dbGaP. **(B)** The 66 members of the tetraspanin and integrin gene families were analyzed for the commonalities that they share within the published literature. The “# Shared rels” is how many of these 66 genes shared the commonality; “Obs/Exp” is the ratio of observed to expected connections, reflecting enrichment of the association; and “Score” is the product of the two (see methods). **(C)** GAMMA prediction charts based on GEO co-expression datasets displaying various predicted gene-function associations in the human cardiovascular system.

In order to find associations between the genes of our interest and cardiovascular phenotypes, we used the database interface Phenotype–Genotype Integrator of NCBI-dbGaP for the investigation. The results of GWAS analysis provided insights into the correlations of integrins and tetraspanins and human diseases on a genome scale (Figure 3A). Integrin gene *ITGA8* has missense single-nucleotide polymorphism (SNP) variant (denoted red in Figure 3A) *rs7895372* associated with cholesterol in an Oceania population (*p* = 6.000 × 10^−6^). Other associations involving missense variations are as follows: *ITGA9* with arterial pressure through *rs146867977* in African Americans (*p* = 7.254 × 10^−6^), *ITGAE* with platelet function tests through *rs2976230* in African Americans (*p* = 1.523 × 10^−5^), and *ITGAM* with platelet function tests through two missense variants, *rs1143678* (*p* = 7.013 × 10^−7^) and *rs1143683* (*p* = 9.980 × 10^−7^), in Europeans. In addition, *ITGA2* has two coding sequence synonymous (cds-syn) variants, *rs1062535* (*p* = 1.096 × 10^−5^) and *rs1126643* (*p* = 1.723 × 10^−5^), that are also associated with platelet function tests in Europeans (denoted in red in Figure 3A).

*TSPAN5, TSPAN15, Cd82, ITGA2*, and *ITGA6* are worth mentioning for their multiple non-coding SNP associations within the introns (denoted in blue in Figure 3A). *TSPAN5* has a dozen of SNPs associated with cholesterol, LDL-cholesterol, and apolipoprotein B in Europeans. *TSPAN15* is correlated with a few different diseases involving thrombosis—venous thromboembolism (71), pulmonary embolism, and stroke in three independent GWAS studies conducted among European or African American populations. *CD82* has one intron SNP *rs730129* that is associated with atherosclerosis and other near-gene or intergenic SNPs that are associated with myocardial infarction, blood pressure, and platelet function tests. In addition to the cds-syn variants mentioned above, *ITGA2* has multiple intron SNPs that are associated with platelet function tests in Europeans, with results coming from the same study (72). *ITGA6* has a potentially versatile role in the cardiovascular system due to containing intron SNPs that correlate with VLDL, echocardiography, platelet function tests, and stroke. There are other near-gene or intergene SNP associations, as shown in green in Figure 3A.

We also investigated *EWI-2* and *EWI-F*, and neither has any association with cardiovascular diseases in human from the analysis. This is probably due to differential expressions between them and their associated tetraspanins in the cardiovascular system. At least in human and mouse large arteries, the expression levels of *EWI-2* and *EWI-F* are much lower than those of *Cd81* and *Cd9*. For example, based on the GTEx database, the numbers of *Cd81* transcripts in human aorta, coronary artery, and tibial artery are ~8.2, 8.8, and 5.5 times as high as the numbers of *EWI-2* transcripts, respectively (data not shown). The association of claudins with cardiovascular diseases did not emerge from the analysis, probably due to less presence of tissues such as the brain and retina, in which tight junction is crucial for vascular integrity, in the datasets.

### GEO Analysis Reveals That Cd9, Cd82, Cd151, TSPAN18, UPK1B, and Most Integrins Are Co-expressed With the Genes Related to Cardiovascular Functions

All human datasets from GEO were processed with specific data mining to extract the co-expressed genes of tetraspanins and integrins in the cardiovascular system. The software IRIDESCENT was used to identify functional commonality between tetraspanins and integrins by grouping their co-expressed genes with terms such as molecules, events, and phenotypes. The 40 most correlated genes with an entire list of tetraspanin and integrin family members were first identified, and then, for each one, common terms associated with the cardiovascular system were identified using literature mining, as described in the methods. Strong commonalities of the integrin and tetraspanin family members highlight their roles in the interactions between ECs and blood cells (e.g., platelets, leukocytes) and between vascular cells and ECM (Figure 3B), further supporting functional connections of tetraspanins to cell adhesion proteins.

We also manually selected those that are closely related to the cardiovascular system. Associations were found between *Cd9, Cd82, Cd151, TSPAN18, UPK1B*, most of the integrins, and various cardiovascular functions (Figure 3C). The most prominent involvements were vessel-related events including microvessels, neovascularization, and vascular development and remodeling, which account for 30% of all associations. Other associations included platelets, heart, ECs, VSMCs, atherosclerosis, fat, and cholesterol. Notably, *Cd9, Cd151, ITGAV*, and *ITGBL1* were associated with two distinct, well-recognized smooth muscle cell markers, SM22alpha (gene symbol: *TAGLN*) and smooth muscle alpha-actin (*ACTA2*), suggesting their potential roles in the regulation of VSMCs.

### Genome-Wide Studies Reveal That ITGAE, Eight Other Integrins, TSPAN5, and Six Other Tetraspanins Are Relevant to Cardiovascular Events in Mice

Table 1 shows the expression levels of relatively highly expressed genes. Results from a variety of genome-wide studies focusing on murine cardiovascular system were deposited in GeneNetwork. Similar to human GWAS, we extracted the data that fell within the spectrum of the genes and phenotypes in which we were interested (Table 2). Among integrins, *ITGA2, ITGA9*, and *ITGAX* were associated with blood pressure, *ITGA2* and *ITGA11* with cholesterol, *ITGA2* and *ITGB6* with platelet mass, and *ITGAE* with platelet count. *ITGA8* and *ITGAE* were associated with heart weight, whereas *ITGA1, ITGA2*, and *ITGA11* were correlated with heart rate. *ITGA1* and *ITGA2* were associated with shortening fraction in echocardiography.

**Table 2:** Associations of integrin and tetraspanin genes with cardiovascular phenotypes in mice.

**Integrins**		**Tetraspanins**	
**Gene**	**Phenotype**	**Gene**	**Phenotype**
*Itgam*	Cardiovascular system, morphology: heart weight	*Tspan5*	Blood chemistry, metabolism: free fatty acid metabolites
*Itgam*	Metabolism, morphology: fat mass	*Tspan5*	Blood chemistry, metabolism: free fatty acids
		*Tspan5*	Cardiovascular system, behavior: run distance
*Itgae*	Cardiovascular system, morphology, aging: heart weight	*Tspan5*	Cardiovascular system, morphology: heart mass
*Itgae*	Cardiovascular system, morphology: heart weight		
*Itgae*	Metabolism, morphology: fat mass	*Tspan11*	Cardiovascular system, behavior: run distance
*Itgae*	Blood chemistry, cardiovascular system: platelet count	*Tspan11*	Cardiovascular system, morphology: heart weight
		*Tspan11*	Metabolism, morphology: fat mass
*Itga2*	Blood chemistry: cholesterol (total)	*Tspan11*	Morphology, metabolism: body fat mass percentage
*Itga2*	Cardiovascular system, behavior: run distance improvement		
*Itga2*	Cardiovascular system, physiology: heart rate	*Tspan9*	Cardiovascular system, behavior: run distance
*Itga2*	Cardiovascular system: shortening fraction	*Tspan9*	Cardiovascular system, physiology: heart rate
*Itga2*	Cardiovascular system: systolic blood pressure	*Tspan9*	Cardiovascular system: shortening fraction
*Itga2*	Blood chemistry, metabolism: total cholesterol	*Tspan9*	Morphology, metabolism: total fat weight
*Itga2*	Central nervous system, cardiovascular system: infarct volume in neocortex following stroke	*Tspan9*	Morphology, metabolism: total subcutaneous adipose tissue weight
*Itga2*	Hematology: mean platelet mass		
*Itga2*	Morphology, metabolism: total fat weight	*Tspan18*	Cardiovascular system, physiology: heart rate
		*Tspan18*	Cardiovascular system: end-diastolic diameter
*Itgax*	Cardiovascular system, physiology: diastolic blood pressure	*Tspan18*	Cardiovascular system: left ventricular mass
		*Tspan18*	Morphology, metabolism: fat gain
*Itga6*	Morphology, metabolism: heart weight	*Tspan18*	Morphology, metabolism: fat
*Itga6*	Morphology, metabolism: total fat weight	*Tspan18*	Morphology, metabolism: total fat weight
*Itga8*	Blood chemistry, cardiovascular system: mean corpuscular hemoglobin	*Upk1b*	Metabolism, morphology: fat mass as percentage of body weight
*Itga8*	Morphology, metabolism: total fat weight	*Upk1b*	Metabolism, morphology: fat mass
*Itga8*	Cardiovascular system, morphology: heart weight	*Upk1b*	Morphology, metabolism: total fat weight
*Itga8*	Cardiovascular system: left ventricular mass		
*Itga8*	Cardiovascular system: left ventricular mass/body weight ratio	*Cd82*	Blood chemistry, cardiovascular system: mean cell hemoglobin concentration
		*Cd82*	Cardiovascular system: systolic blood pressure
*Itgb6*	Hematology: mean platelet mass		
*Itga1*	Cardiovascular system, physiology: heart rate		
*Itga1*	Cardiovascular system: shortening fraction		
*Itga1*	Morphology, metabolism: total fat weight		
*Itga11*	Cardiovascular system, physiology: heart rate		
*Itga11*	Blood chemistry, metabolism: total cholesterol		
*Itga11*	Morphology, metabolism: total fat weight		
*Itga9*	Cardiovascular system: systolic blood pressure		

*The listed associations between genes and traits are the significant ones, i.e., the p value of the sample's correlation coefficient r was <0.05. Phenotypes are presented as the traits. For example, “Morphology” denotes a class of traits involving general biologic parameters of mice, such as heart mass, heart weight, and fat weight*.

As for tetraspanins, *TSPAN5* was associated with free fatty acids in the blood, while *TSPAN11* was associated with heart weight, *TSPAN9* with end-diastolic diameter in echocardiography, and *TSPAN18* with heart rate. *Cd82* was associated with blood pressure.

Other loose associations (i.e., the ones with relatively larger *p* values) with cardiovascular phenotypes include the following: *ITGA2* with the infarct size in neocortex after experimental stroke; *ITGA2, TSPAN5, TSPAN9*, and *TSPAN11* with run distance performance; and *UPK1B* with fat, fat mass, or fat weight.

### Based on the Data From Both Species, TSPAN5 and ITGA8 Are Associated With Lipid Metabolism, Cd82 and ITGA9 With Blood Pressure, and ITGA2, ITGAE, and ITGB6 With Platelet Functions

**Figure 4:**
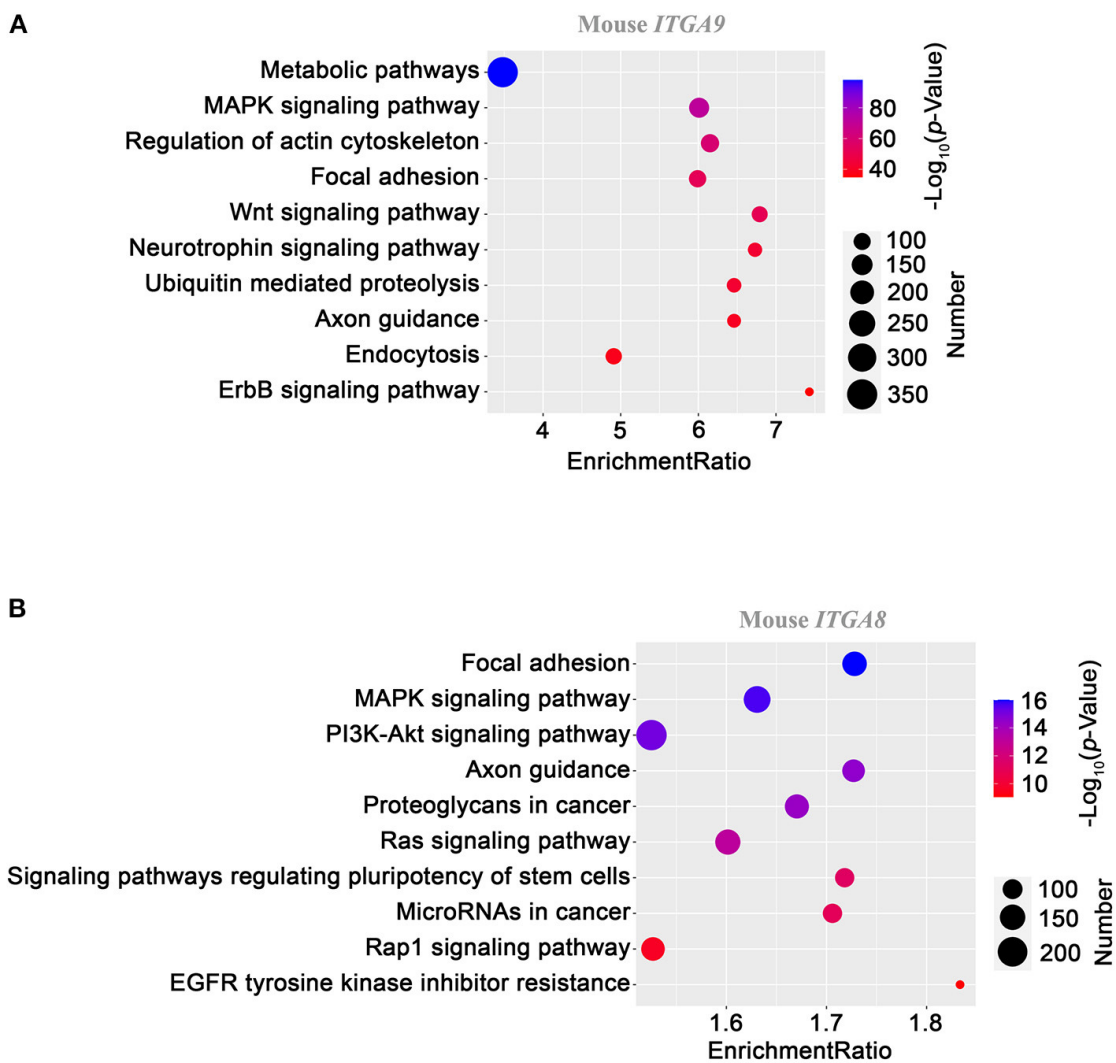
KEGG pathway enrichment analysis on ITGA9 **(A)** and ITGA8 **(B)** using gene correlation data from mouse aortas. Gene set enrichment analysis was performed as described in the *Methods* section. The dot size presents the number of enriched genes with the indicated pathway, and the dot color codes the level of –log_10_ of the *p* value. The enrichment ratio is defined as the number of observed genes divided by the number of expected genes in a KEGG category.

We choose *ITGA9* for further analysis. At the protein level, more than 90% of sequences identity between human and mouse *ITGA9* make them highly comparable. The KEGG pathway-based gene set enrichment analysis using the co-expression data from mouse aorta suggested that *ITGA9* is involved in metabolism, MAPK signaling, actin cytoskeleton, focal adhesion, and Wnt signaling. Figure 4A shows the top 10 enriched pathways according to the adjusted *p* value.

A similar analysis was applied to *ITGA8*, given its potential involvement in cholesterol metabolism. Focal adhesion, MAPK signaling, and PI3K-Akt signaling are the top three pathways (Figure 4B), reminiscent of the readouts from *ITGA9* analysis.

## Discussion

High-throughput data, such as the data of genome, transcriptome, proteome, metabolome, and phenome, not only convey massive amounts of insightful information but also contain considerable noise (73). The noise can be caused by, for example, factors such as biological variation or statistical variance. Here, we have integrated the analyses of human and mouse data to determine possible significant “signals” by matching the readouts with the area of our research interest. Such a comprehensive approach in principle likely provides better conclusion for establishing a close relationship between a molecular change and a pathophysiological event.

Both integrins and tetraspanins exhibit markedly diverse expression levels across their family spectra in the cardiovascular system, so do the expression levels of a given member of the families across different human tissues. Some of them such as *Cd151* and *ITGA8* are highly expressed in arteries, probably to some extent reflecting their functional connection, although tissue specificity of a gene does not necessarily correlate with the gene expression level in the tissue. CD151, as stated earlier, is a modulator of vascular functions, and integrin α8 is a crucial regulator of smooth muscle cells (74). As shown in the expression heatmap, *ITGA9* has a moderate expression level in human aorta compared with other integrins, but it exhibits an overall low-expression profile across all other tissues. A previous study showed that the abundance of a protein in a certain state is the result of optimization of the cost vs. benefit (75). Also, the expression of mRNA is not always correlated with the expression of protein (76). Hence, *ITGA9* may affect vascular function like the regulation of blood pressure without having a high mRNA expression profile in the cardiovascular system.

Moreover, we found many intronic variants in the human GWAS database. *TSPAN5*, for example, has various intronic SNPs correlated with cholesterol or “cholesterol, LDL.” A previous study (77) and a literature review (78) led to our belief that these introns may contain transcriptional regulatory elements, such as DNA methylation loci, and therefore may alter the quantity or feature of the transcripts of this gene. For example, the intronic SNPs could be transcriptional regulatory elements to alter the RNA splicing of *TSPAN5*. Similarly, the intergenic variants may influence the phenotype through transcriptional modifications. Previous studies showed that cds-syn variants may cause the instability of the mRNA or even the alteration of protein conformation (79, 80). The associations of missense variants are easier to understand, since the changes in protein sequences could directly lead to functional loss or gain (81, 82). The statistical significance of a genome association was generally determined by a *p* value that is <5 × 10^−8^ (83). None of the exon variants that we found were significant following this standard, but we still considered the association to be not random and be suggestive if the same association was found in mice. Some of the variants located in introns meet the criteria: for instance, an association between an SNP within *TSPAN15* (*rs78707713*) and venous thromboembolism has a *p* value of 2 × 10^−16^. Thus, it is likely that this gene is involved in the pathogenesis of venous thromboembolism, either as a cause or a result.

The data organization and processing in GeneNetwork are similar to those in the human databases that we used, and complement the results from the human databases. *ITGA9* has associations with the same phenotype, i.e., blood pressure, in both species. In humans, the relevant variant, *rs146867977*, is located within the exon of *ITGA9* and causes a missense alteration. In mice, blood pressure as a trait was found to have a maximum association “peak” that falls within the *ITGA9* gene sequence. *ITGA9* has a very long genomic DNA sequence in both humans and mice (~360,000 and ~290,000 bp, respectively). The chance of having a random association, therefore, increases. A previous study found that a different SNP of *ITGA9* is associated with hypertension (*rs7640747, p* = 4.8 × 10^−7^) (84), consistent with our prediction. Interestingly, the pathway analysis reflected what has already been reported, assuring us of the reliability of our analyses. For instance, MAP kinase and downstream molecules are indeed activated by integrins (85, 86) and so are actin cytoskeleton (87), Wnt signaling (88), and focal adhesion (89). Of course, *in vivo* experiments are needed to determine whether integrin α9 actually regulates blood pressure.

Furthermore, we have combined transcriptional correlations derived from GEO expression datasets with a literature-mining tool, IRIDESCENT, to predict gene-function associations using a “guilt by association” approach (63). The ability of the approach to predict a number of already-established literature commonalities lends additional plausibility to the unestablished ones. Predicted associations with blood vessels, for example, were shown by earlier studies to rely on integrins and tetraspanins in biological processes such as angiogenesis (49, 90).

This bioinformatics study has several limitations. First, we rely upon deposited public data, which is often incompletely annotated and of varying quality both on the individual experiment level (e.g., some studies may be statistically underpowered) and the aggregate level (e.g., most studies are small scale, but some are very large, creating the possibility of batch effects). Thus, genes identified with the highest confidence will tend to be those with larger transcriptional changes, and the relevance of a gene to cardiovascular biology on a different level (e.g., translation, protein–protein interactions, etc) will not be detectable with these methods. Another limitation of data mining for cardiovascular diseases is that, unlike cancer-related databases, data sources and analysis tools are limited currently. Thus, we cannot make comparisons across platforms. In addition, as the human data was not stratified by age, gender, or ethnicity, we may not understand the data in great depth or may miss new associations. Moreover, GWAS-based phenotypes may be indirect or non-specific. For example, a mutation affecting energy production or waste disposal would likely affect many things, including cardiovascular function. In part, we attempt to compensate for this limited perspective with the transcriptional network analysis, which will tend to weight specific associations (e.g., cardiomyocytes) higher than general ones. Given very limited availability of mouse GWAS data (one dataset from mouse vs. hundreds of datasets from human), our observation of the little overlap in gene–phenotype associations between humans and mice could reflect an incomplete view. Nonetheless, the databases used here are major repositories with high-quality uniform datasets that combine numerous large-scale studies. New techniques such as single-cell RNAseq, when widely applied to the cardiovascular research field, will bring more details into the association studies, adding a dimension of marker-specific subpopulations and subgroups for more thorough analysis (91). Apparently, the conclusions from all bioinformatics analyses need to be confirmed by experimental approaches, so do the observations made by this bioinformatics study, in order to establish causal relationships between TEM genes and cardiovascular pathophysiology.

## Conclusion

On the basis of combined analyses on human and mouse data, our study identified and predicted that *ITGA9* is highly relevant to cardiovascular diseases among integrins and tetraspanins and promising for further experimental studies, although this integrin was already found to support lymphatic and venous valve formation and thrombosis (92–94). Other highlighted associations are *TSPAN5* and *ITGA8* with lipid metabolism, *Cd82* with blood pressure, and *ITGA2, ITGAE*, and *ITGB6* with platelet functions. This work provides an example of using integrated bioinformatics approaches to predict the links of structurally and functionally related genes and proteins to given diseases in a comprehensive and systematic manner.

## Data Availability Statement

The datasets presented in this study can be found in online repositories. The names of the repository/repositories and accession number(s) can be found in the article/supplementary material.

## Author Contributions

GS, JC, JW, and FX performed analysis and wrote the manuscript. YD wrote the manuscript. LL, YW, and D-wW supervised the study. XZ conceived, designed, and supervised the study and wrote the manuscript. All authors contributed to the article and approved the submitted version.

## Conflict of Interest

The authors declare that the research was conducted in the absence of any commercial or financial relationships that could be construed as a potential conflict of interest.

## Abbreviations

cds-syn: coding sequence synonymous
ECs: endothelial cells
ECM: extracellular matrix
eQTL: expression quantitative trait loci
FEVR: familial exudative vitreoretinopathy
GEO: Gene Expression Omnibus
GWAS: genome-wide association studies
GTEx: Genotype-Tissue Expression
KEGG: Kyoto Encyclopedia of Genes and Genomes
SNP: single-nucleotide polymorphism
TEM: tetraspanin-enriched microdomain
VSMC: vascular smooth muscle cell.
